# External validation of the relative fat mass (RFM) index in adults from north-west Mexico using different reference methods

**DOI:** 10.1371/journal.pone.0226767

**Published:** 2019-12-31

**Authors:** Alan E. Guzmán-León, Ana G. Velarde, Milca Vidal-Salas, Lucía G. Urquijo-Ruiz, Luz A. Caraveo-Gutiérrez, Mauro E. Valencia

**Affiliations:** Department of Chemical-Biological Sciences, University of Sonora, Sonora, México; Kennesaw State University, UNITED STATES

## Abstract

**Background:**

Analysis of body composition is becoming increasingly important for the assessment, understanding and monitoring of multiple health issues. The body mass index (BMI) has been questioned as a tool to estimate whole-body fat percentage (FM%). Recently, a simple equation described as relative fat mass (RFM) was proposed by Woolcott & Bergman. This equation estimates FM% using two anthropometric measurements: height and waist circumference (WC). The authors state that due to its simplicity and better performance than BMI, RFM could be used in daily clinical practice as a tool for the evaluation of body composition. The aim of this study was to externally validate the equation of Woolcott & Bergman to estimate FM% among adults from north-west Mexico compared with Dual-energy X-ray absorptiometry (DXA) as an alternative to BMI and secondly, to make the same comparison using air displacement plethysmography (ADP), Bioelectrical Impedance Analysis (BIA) and a 4-compartment model (4C model).

**Methods:**

Weight, height and WC were measured following standard procedures. The RFM index was calculated for each of the 61 participating subjects (29 females and 32 males, ages 20–37 years). The RFM was then regressed against each of the four body composition methods for estimating FM%.

**Results:**

Compared with BMI, RFM was a better predictor of FM% determined by each of the body composition methods. In terms of precision the best equation was RFM regressed against DXA (y = 1.12 + 0.99 x; R^2^ = 0.84 p<0.001). Accuracy (represented by the closeness to the zero-intercept) was 1.12 (95% CI: -2.44, to 4.68) and thus, not significantly different from zero. For the rest of the methods, precision in the prediction of FM% was improved compared to BMI, with significant increases in the R^2^ and reduction of the root mean squared error (RMSE). However, the intercepts of each regression did not show accuracy since they were different from zero, for ADP: -9.95 (95%CI: -15.7 to -4.14), for BIA: -12.6 (95%CI: -17.5 to -7.74) and for the 4C model: -13.6 (95%CI: -18.6 to -8.60). Irrespectively, FM% measured by each of the body composition methods was higher for DXA than the other three methods (p<0.001).

**Conclusions:**

This external validation proved that the performance of the RFM equation used in this study to estimate FM% was more consistent than BMI in this Mexican population, showing a stronger correlation with DXA than with the other body composition methods.

## Introduction

The body mass index (BMI) has been used extensively in epidemiological and clinical studies to classify overweight and obesity. BMI is the weight in kilograms divided by the square of height in meters and is strictly a measure of body size. The conventional World Health Organization (WHO) classification for body weight status is underweight (<18.5 kg/m^2^), normal weight (18.5–24.9 kg/m^2^), overweight (25–29.9 kg/m^2^), and obese (>30 kg/m^2^) [1].

Overweight and obesity are conditions that substantially increase morbidity from hypertension, dyslipidemia, type 2 diabetes, coronary heart disease and other pathologies [2]. Higher body weights are also associated with an increase in all-cause mortality [3]. BMI has been used to classify underweight and overweight [4] and used together with physical activity to diagnose chronic energy deficiency [5]. Also, BMI has been applied in childhood and puberty (standardized by age and sex) to define malnutrition and obesity [6]. However, BMI does not consider the distribution of fat mass (FM) and fat free mass (FFM) [7–10]. People with identical BMI can vary widely in percent body fat, which can lead to misclassification of body-fat defined obesity [10].

Recently, a very simple equation described as relative fat mas (RFM) was proposed by Woolcott & Bergman [11]. This equation estimates whole-body fat percentage (FM%) using two anthropometric measurements: height and waist circumference (WC). The National Health Examination Survey (NHANES) 1999–2004 data (n = 12,581) was used for model development and NHANES 2005–2006 data for model validation. Dual-energy X-ray absorptiometry (DXA) was used as the reference method. Data from adult individuals 20 years of age and older were analyzed. Compared with BMI, RFM better predicted FM%: R^2^ = 0.84; root mean squared error (RMSE) = 3.51% against BMI: R^2^ = 0.36; RMSE = 7.04% [11]. The authors state that due to its simplicity and overall better performance than BMI among women and men RFM could be used in daily clinical practice as a tool for the evaluation of body composition in healthy or ill patients, as well as to monitor changes in FM%.

Although, there is evidence showing that DXA as a reference method tends to overestimate the FM%, overall differences between this and other reference methods are relatively small [12].

The aim of this study was to externally validate the equation of Woolcott & Bergman to estimate FM% among adults from north-west Mexico compared with DXA as an alternative to BMI and secondly, to make the same comparison using air displacement plethysmography (ADP), Bioelectrical Impedance Analysis (BIA) and a 4-compartment model (4C model).

## Material and methods

### Study population

Our mestizo population in the State of Sonora in Northwest Mexico, comes from regional indigenous groups and Europid population. Genomic diversity has been studied in the Mexican Mestizo populations [13] as well as admixtures and population structure studies [14]. European colonization in Mexico produced a complex biological admixture, mainly between Native Americans, Spaniards and African slaves [14]. Mestizos are a result of this process [15]. Indigenous population diminished through time due to epidemics and recovered some time later, although some ethnic groups disappeared completely [16]. Further, other events from the 16^th^ to the 18^th^ century like the discovery of silver mines in Northern Mexico, contributed to the admixture [16,17].

In the early 1800s, the north western states like Sonora, Mexico, received European migrants from France, Italy, Germany, among others with interest in agricultural development [18–20]. Many of the surnames originating from these countries still prevail. A study of genomic diversity [13] analyzed 300 unrelated self-identified Mestizo individuals from 6 States located in geographical distant regions of Mexico, including the northern State of Sonora. Based on heterozygosity (HET) analysis, they found that among Mexicans, the northern populations of the States of Sonora and Zacatecas, had the highest HET values, 0.287 and 0.286, respectively; suggesting more genetic diversity, while the sample of Zapotecan Amerindians, had the lowest HET, with a value of 0.229, as could be expected for an isolated population. Moreover, looking at private alleles the results correlated with the observation that Northern Mexican subpopulations, Sonora and Zacatecas had the highest European ancestral contribution [13].

Moreover, the total population of State of Sonora, Mexico, reported in the Census of 2010 was 2.662 million inhabitants, while the indigenous population was 137,560 (5.16%), and only, 60,611 spoke their native languages or dialects (2.3%). They come from 9 ethnic groups: Mayo (47.2%), Yaqui (26.5%), migrants from other indigenous communities in Mexico (21.8%), Papago (1.4%), Guarijío (1.1%), Comca’ac (0.76%), Pima (0.71%), Cucapá (0.34%) and Kikapú (0.06%) [21].

The conceptual frame of marginalization Indexes is given by Mexico’s National Council of Population [22]. It includes socioeconomic dimensions such as education, housing, population distribution and income. The average marginalization index for all participants was calculated based on their current address, including street, number and sector. This was cross-referenced to their Basic Geostatistical Area (AGEB) based upon the National Census of 2010, reported by INEGI [23].

The study was conducted at the Body Composition Laboratory, University of Sonora, in the city of Hermosillo, Mexico. Thirty-two male and 29 female Mexican mestizo, subjects, ages 20–37 years were selected as an opportunity sample from a group of subjects of a body composition study under the responsibility of the corresponding author. Subjects were recruited from September 2014 to December of 2015. Participants were instructed not to drink water or consume food, 2 hours prior to the time of measurement (10 AM to 2 PM).

Subjects were either students or employees working in administration, secretarial or professional positions. Physical activity was not a selection criterion, however, they reported mostly low to moderate physical activity. Only subjects that were above 20 years of age and reported being healthy and not taking medications that could alter their body composition status, such as thyroid hormones, glucocorticoids, diuretics or anti-obesity drugs were included. An experienced operator performed anthropometric measurements and body composition parameters. Participants who did not complete all measurements were excluded from the analysis. All subjects signed a written informed consent before any measurement was taken. Confidentiality was strictly maintained throughout the study and data collection forms were safely stored.

### Ethics statement

The protocol was approved by the Bioethics and Research Committee of the Department of Medicine and Health Sciences of the University of Sonora, in Hermosillo, Sonora, Mexico. The investigators complied with applicable requirements of the declaration of Helsinki [24].

### Anthropometric measurements

Body weight for BMI and body composition variables was obtained by the BOD POD’s electronic scales during the measurement of body volume. Height was measured to the nearest 0.1 cm with a Harpenden stadiometer (Holtain Limited, United Kingdom; 600 mm to 2100 mm), with no shoes. Feet were placed with the tips slightly separated, body completely supported in the stadiometer, in the Frankfurt plane. The BMI was calculated as weight (kg)/[height(m)]^2^. WC was measured using a retractable Gülick glass fiber tape (1500 ± 1 mm) with a tension meter at the level of the umbilical scar in a standing position and after exhaling [25].

### Body composition measurements

#### Dual-energy X-ray absorptiometry (DXA)

Bone mineral content (BMC), FM and FFM (kg) were measured by DXA (QDR series, Hologic Discovery A, Hologic, Inc. Bedford, Ma, EUA). Additionally, each DXA scan provided regional measurements of FM for arms, legs and abdominal segment.

Subjects were exposed to a micro-dose of radiation of 4.5 microsieverts (uSv), equivalent to 12 hours of direct radiation at sea level. Scans were performed while subjects were wearing light indoor clothing (T-shirts and shorts) and with no metal objects. Subjects were placed in supine position on the scanner bed with the arms slightly apart from the sides, the tip of the feet slightly inwards, for a lapse of 3 minutes to complete the scan. Measurements were performed under the supervision of a certified technician (Mexican Association of Bone and Mineral Metabolism).

#### Bioelectrical impedance analysis (BIA)

Total body water (TBW) was estimated by bioimpedance prediction equation for Caucasian adults proposed by Kushner & Schoeller based on deuterium oxide (D_2_O) dilution in obese and non-obese subjects [26]. Their study developed two specific equations for calculating TBW. For males: D_2_O –TBW _Liters_ = 0.396 (Height^2^_cm_ / Resistance _ohms_) + 0.143 Weight _kg_ + 8.399 (R^2^ = 0.976; SEE = 1.662_liters_), and for females: D_2_O- TBW _liters_ = 0.382 (Height^2^_cm_ / Resistance_ohms_) + 0.105 Weight_kg_ + 8.315 (R^2^ = 0.95; SEE = 1.509_Liters_).

From predicted TBW, FFM was calculated assuming a hydration factor of 0.73 [27] and FM from the difference with body weight.

Additional to weight and height, resistance (R) and reactance (Xc) were measured after 5 minutes of rest using an RJL Quantum-X bioimpedance meter (RJL Systems). Subjects were instructed not to consume alcoholic beverages 24 hours before the measurement nor to eat or drink 2 hours before the measurement.

#### Measurement of body volume

Body volume was measured using air displacement plethysmography (BOD POD Body Composition System, Life Measurement Instruments, Concord, Calif. USA). The BOD POD has a single structure containing two chambers, electronically controlled by a servo system, produces pressure fluctuations in both chambers which are used to assess the total body volume. The system has been described in detail elsewhere [28,29]. Each subject was weighed on a calibrated scale to a resolution of 0.01 kg.

A two-point calibration was then performed as baseline, with the chamber empty and using 50 liters as the calibration for the cylinder. The subject entered the chamber in a tight swimming suit and cap and sat down for the volume measurements which were done in duplicate for each subject. Lung volume corrections were made based on the estimation of the BOD POD software. From the corrected body volume and body mass, body density was obtained, and percent body fat was calculated using Siri’s equation and BOD POD’s software. The reproducibility of this system has been reported earlier [29].

#### Four compartments body composition model (4C model)

The 4C model was also used to estimate FM using Selinger’s equation [30] as follows: %Fat = (2.747/D_b_− 0.714W + 1.146B − 2.0503) X100. Where D_b_ is body density; W = total body water as a fraction of body weight; B = osseous mineral as a fraction of body weight. Total bone mineral was calculated by multiplying BMC obtained from DXA by 1.22 [31]. The FFM was estimated from the difference between body mass and FM.

### Relative fat mass (RFM)

The relative fat mass concept according to Woolcott & Bergman [11] was calculated as, RFM = 64 –[20 x (height/waist circumference) both in meters] + (12 x sex). Sex = 0 for male and 1 for female. This RFM equation, which was validated against DXA as an estimator of FM% was applied to our subjects and compared to the percentage of fat mass measured by each of the body composition methods: DXA, ADP, BIA and the 4C model.

### Statistical analysis

Med Calc Software bvba, Version 18.2.1, was used for analysis. Descriptive statistics include the analysis of central tendency variables and dispersion. All variables were analyzed for normality of distribution using the Shapiro-Wilk test. Normally distributed variables are presented as means ± standard deviation (SD) and non-normally distributed variables are reported as medians and interquartile range (IQR). However, all variables of physical and anthropometric characteristics are reported as means, medians, IQR and range. The comparison of FM% measured by the different methods of body composition was done by the Kruskal Wallis non-parametric analysis of variance and medians compared by a pairwise test of subgroups according to Conover [32].

The RFM and BMI were each regressed on measured percent fat mass by DXA, ADP, BIA and the 4C model. Precision of the individual techniques to estimate FM% is given by the model R^2^ and the RMSE from each regressions of the body composition methods. Precision was also calculated by Pearson’s correlation coefficient (r) that measures how far each observation deviates from the best-fit line. By the same token, a measure of accuracy is the deviation of the best-fit line with respect to the 45° line through the origin [33,34]. Significant deviations from these zero intercepts for each regression of BMI and RFM pairs with respect to DXA, ADP, BIA and the 4C model were determined from a t test and the 95% CI.

## Results

Demographic characteristics of participants are presented on Table 1, showing a low proportion of individuals that do not have access to adequate and basic socio demographic indicators. Only 3.28% classified as highly marginalized status, while the great majority, 86.9% corresponded low or very low marginalization, that could correspond to middle or upper middle-income levels [35].

**Table 1:** Percent and 95% CI of individuals that do not have access to the following conditions.

	All n = 61	Female n = 29	Male n = 32
**Primary Education**	1.84 1.56 to 2.18	1.60 1.25 to 2.06	2.09 1.66 to 2.63
**Secondary Education**	17.6 14.7 to 21.8	14.9 11.3 to 19.7	20.4 16.1 to 26.0
**Health services**	20.4 19.0 to 21.8	19.8 17.5 to 22.3	20.9 19.4 to 22.5
**Running water**	0.97 0.60 to 1.56	0.66 0.33 to 1.33	1.29 0.66 to 2.52
**Sewage systems**	1.39 0.91 to 2.13	1.04 0.54 to 2.02	1.70 0.95 to 3.05
**Toilet facilities**	1.38 0.90 to 2.12	1.04 0.54 to 2.02	1.69 0.95 to 3.02
**Cement or tiled floors**	1.22 0.91 to 1.65	1.00 0.64 to 1.59	1.50 1.01 to 2.22
**Refrigerator**	1.22 0.95 to 1.56	0.90 0.62 to 1.32	1.59 1.16 to 2.18
**Automobile**	19.5 15.7 to 24.2	15.3 10.9 to 21.4	24.4 18.7 to 31.7
**Degree of urban marginalization**			
**High, %**	3.28	3.45	3.13
**Medium, %**	9.84	3.45	15.6
**Low, %**	24.6	13.8	34.4
**Very low, %**	62.3	79.3	46.9

The study group included 61 subjects (29 females and 32 males) aged 20–37 years. Table 2 shows the physical and anthropometric characteristics of the population. Body weight, height, and BMI in both groups ranged from 43.7–140 kg, 146–185 cm and 17.4–49.6 kg/m^2^, respectively. In the case of WC and RFM, the values for both groups were 64.5–125 cm and 16.6–49.2%. Median and IQR body composition variables by DXA for men and women together were FM 19.6 (15.4 to 25.7) kg, FFM 42.5 (36.8 to 49.7) kg, BMC 2.12 (1.91 to 2.40) kg. The proportion of individuals with a BMI ≥ 25 kg/m^2^ was 32.8% (21.3% ≥ 25 <30 and 11.5% ≥30 kg/m^2^).

**Table 2:** Physical and anthropometric characteristics.

	All n = 61	Female n = 29	Male n = 32
**Age (years)**			
Mean	24.3	25.7	23.1
Median	23.0	25.0	22.5
IQR	22.0 to 25.3	22.0 to 29.3	22.0 to 24.5
Range	20.0–37.0	20.0–37.0	20.0–28.0
**Body weight (kg)**			
Mean	66.8	65.6	67.8
Median	64.2	61.1	68.2
IQR	56.0 to 73.2	53.5 to 70.2	58.3 to 75.6
Range	43.7–140	43.7–140	44.4–96.5
**Height (cm)**			
Mean	164	160	167
Median	164	161	167
IQR	160 to 169	157 to 164	165 to 172
Range	146–185	146–170	154–185
**BMI**^a^			
Mean	24.7	25.3	24.0
Median	23.6	23.3	24.0
IQR	21.5 to 26.0	21.5 to 26.8	21.4 to 25.6
Range	17.4–49.6	18.4–49.6	17.4–35.1
**Waist circumference (cm)**			
Mean	83.9	83.3	84.4
Median	83.0	81.0	84.5
IQR	76.0 to 88.4	74.6 to 87.7	76.0 to 89.0
Range	64.5–125	64.5–125	69.5–105
**FM (kg)**^b^			
Mean	21.6	26.1	17.5
Median	19.6	21.2	17.8
IQR	15.4 to 25.7	18.0 to 28.1	12.0 to 21.0
Range	7.47–68.4	15.2–68.4	7.47–32.4
**FFM (kg)**^b^			
Mean	44.3	38.6	49.4
Median	42.5	38.6	48.7
IQR	36.8 to 49.7	34.3 to 41.6	44.8 to 52.7
Range	27.5–67.5	27.5–67.4	36.2–67.5
**BMC (kg)**^b^			
Mean	2.16	2.06	2.26
Median	2.12	2.01	2.23
IQR	1.91 to 2.40	1.87 to 2.23	1.94 to 2.48
Range	1.48–3.14	1.54–2.87	1.48–3.14
**RFM (%)**^c^			
Mean	30.1	36.9	23.9
Median	29.0	37.4	23.4
IQR	23.3 to 36.2	33.2 to 38.7	21.5 to 26.0
Range	16.6–49.2	28.2–49.2	16.6–32.5

Abbreviations: BMC, bone mineral content; BMI, body mass index; FFM, fat free mass; FM, fat mass; IQR, interquartile range; RFM, relative fat mass.

^a^BMI is weight in kilograms divided by the square of height in meters.

^b^BMC, FFM and FM were measured by DXA.

^c^RFM was calculated by Woolcott & Bergman’s equation [64–(20 x (height/waist circumference)) + (12 x sex)]; height and waist circumference are expressed in meters and sex = 0 for males and 1 for female [11].

**Fig 1:**
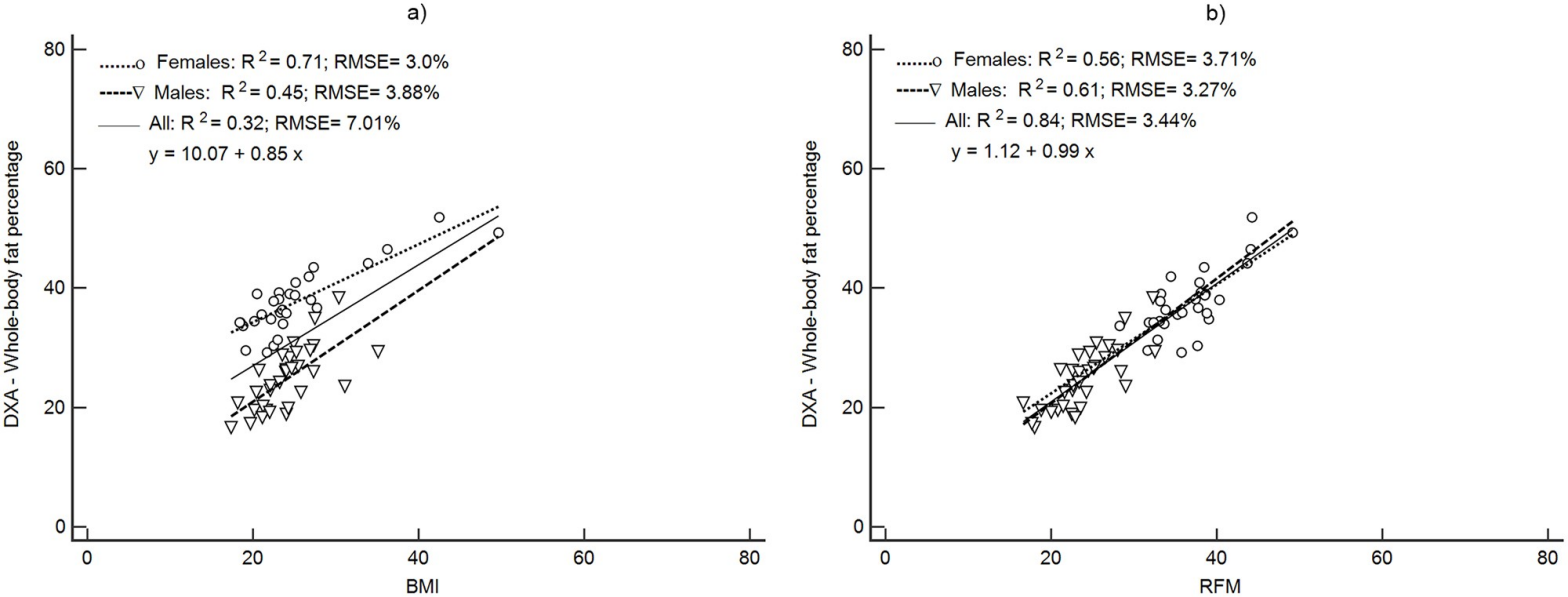
Prediction of FM% from BMI and RFM using DXA as a body composition method. Abbreviations: BMI, body mass index; DXA; RFM, relative fat mass; RMSE, root mean squared error.

**Fig 2:**
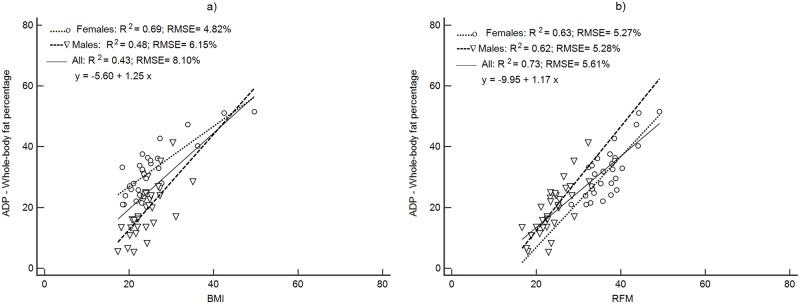
Prediction of FM% from BMI and RFM using ADP as a body composition method. Abbreviations: ADP, air displacement plethysmography BMI, body mass index; RFM, relative fat mass; RMSE, root mean squared error.

**Fig 3:**
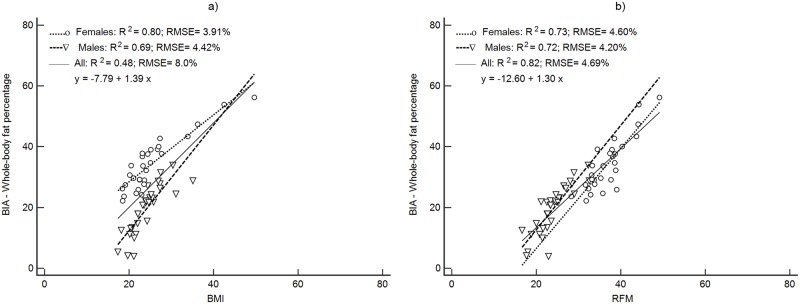
Prediction of FM% from BMI and RFM using BIA as a body composition method. Abbreviations: BIA, bioelectrical impedance analysis, body mass index; RFM, relative fat mass; RMSE, root mean squared error.

**Fig 4:**
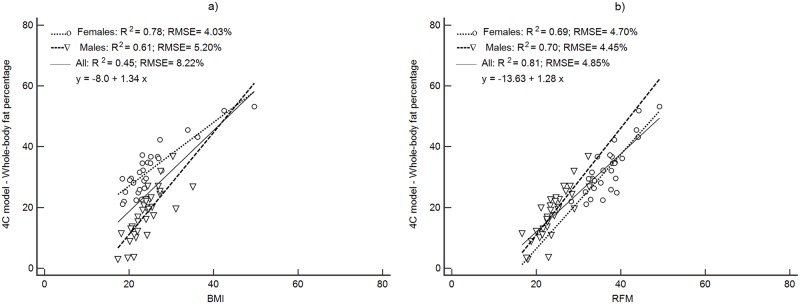
Prediction of FM% from BMI and RFM using 4C model as a body composition method. Abbreviations: BMI, body mass index; RFM, relative fat mass; RMSE, root mean squared error; 4C model, 4-compartment model.

Figs 1–4 depict the prediction of FM% by RFM based on height/waist and by BMI using linear regression. Plots are presented separately by sex and with both groups together. All regression plots show that RFM compared to BMI is a better predictor of FM% for DXA, ADP, BIA and the 4C model. For all body composition methods, the prediction of FM% by RFM based on the RMSE was better with male and female together than separately. The increase in proportion of the variability explained (R^2^) with both groups together was 26% (Fig 1b) for DXA, 11% (Fig 2b) for ADP, 10% (Fig 3b) for BIA and 12% (Fig 4b) for the 4C model.

The RFM calculated by Woolcott & Bergman’s equation [11] and BMI were regressed against each of the body composition methods for predicting FM%, DXA, ADP, BIA and the 4C model. Fig 1 shows the regression equations for FM% measured by DXA versus BMI and RFM. Compared with BMI, RFM was a better predictor of FM% determined by each of the body composition methods. In terms of precision, the best equation was RFM regressed against DXA (y = 1.12 + 0.99 x; R^2^ = 0.84 p<0.001) (Fig 1b). Moreover, WC had a higher correlation with abdominal fat mass from DXA (r = 0.81; p<0.00001) compared to regional fat mass from arms (r = 0.72; p<0.0001), legs (r = 0.54; p<0.001) and total fat mass (r = 0.72; p<0.0001).

For the rest of the methods, precision in the prediction of FM% was improved compared to BMI, with significant increases in Pearson’s r (p<0.001), increases the R^2^ and reduction of the RMSE (Table 3).

**Table 3:** Linear regression equation comparison of BMI and RFM against FM% obtained from the body composition methods.

Method		Precision				Accuracy	
		Pearson´s r	p-value	R^2^	RMSE (%)	Intercept	p-value
**DXA** **(FM%)**	BMI	0.51	<0.001	0.32	7.01	10.1 (1.85 to 18.3)	0.0172
	RFM	0.91		0.84	3.43	1.12 (-2.44 to 4.68)	0.5328
**ADP** **(FM%)**	BMI	0.66	<0.01	0.43	8.10	-5.60 (-15.1 to 3.89)	0.5328
	RFM	0.85		0.73	5.60	-9.95 (-15.7 to -4.14)	0.0011
**BIA**^**a**^ **(FM%)**	BMI	0.49	0.001	0.49	8.00	-7.80 (-17.2 to 1.60)	0.1019
	RFM	0.82		0.82	4.69	-12.6 (-17.5 to -7.74)	<0.0001
**4C model** **(FM%)**	BMI	0.45	<0.001	0.45	8.22	-7.99 (-17.62 to 1.64)	0.1020
	RFM	0.90		0.81	4.85	-13.63 (-18.6 to -8.60)	<0.0001

Abbreviations: ADP; air displacement plethysmography; BIA, bioelectrical impedance analysis; BMI, body mass index; DXA; dual- energy X-ray absorptiometry; RFM, relative fat mass; RMSE, root mean squared error; 4C model, 4-compartment model. Regression equation comparisons by body composition methods, was done using a Fisher’s Z transformation for correlation coefficient´s (Pearson´s r). Precision: improvement of precision is given by the significant increase in Pearson’s r, with simultaneous decrease in RMSE %. Accuracy: improvement of accuracy is given by a non-significant difference from the zero intercept of each regression.

^a^BIA was estimated by bioimpedance prediction equation proposed by Kushner & Schoeller [26].

Accuracy (represented by the closeness to the zero-intercept) of RFM regressed against DXA was 1.12 (95% CI: -2.44, to 4.68) and thus, not significantly different from zero (Fig 1b). The intercepts of each regression of the other methods did not show accuracy since they were significantly different from zero (Table 3). The intercepts and 95% CI were: -9.95 (95%CI: -15.7 to -4.14), -12.6 (95%CI: -17.5 to -7.74) and -13.6 (95%CI: -18.6 to -8.60) for ADP, BIA and the 4C model respectively (Table 3).

The analysis of FM% measured by the different body composition methods were not different for BIA, ADP and the 4C model with medians (IQR) of 26.6 (21.2–33.8), 24.5 (16.8–32.8) and 24.9 (17.3–32.1), respectively. However, FM% by DXA (30.4, 23.7–37.7) was higher (p<0.001).

## Discussion

This external validation process was a way to look at Woolcott and Bergman’s RFM algorithm and how it would hold, when our subjects were measured, principally by DXA, and also, ADP, BIA and the 4C model, considering possible differences in body composition of our group of subjects from north-west Mexico.

We found that, compared with BMI, RFM was a better predictor of FM% determined by each of the body composition methods. Further, the best equation was RFM regressed against DXA. Historically, the prediction of FM and FFM has been implemented by using anthropometric measurements validated by reference methods, such as the ones used in our study. Some of the most widely used prediction equations are those of Durnin-Womersley [36] based on one to four skinfolds measured in 16-72-year-old men and women in Glasgow, Scotland, in 1974 and validated by hydro densitometry. Further, the method has been applied in the estimation of FM% in diverse ethnic groups such as Caucasians, Latinos, Asians and Africans in 111 countries. Nowadays, however, we suggest that body fat distribution would be better represented by equations that consider other fat deposition sites such as the abdominal region, measured at the waist level, and validated by DXA or MRI as this is likely to have more relevance to the metabolic syndrome, type 2 diabetes and cardiovascular diseases [37–39].

The study by Woolcott and Bergman [22] aimed to identify a simple anthropometric equation for adults to test the validity of the height-to-waist ratio as an indicator of FM%. The NHANES 1999–2004 data (n = 12581) used for the model development and NHANES 2005–2006 data (n = 3456) for model validation included Mexican-American, European-American, and African-Americans. Our analysis is an external validation of the RFM estimator of whole-body fat in this group of adults from north-west Mexico, despite the limitation of a smaller sample size, lower body weight, BMI and proportion of overweight and obesity.

Like Woolcott and Bergman [11], we found a significant improvement in the ability of RFM to predict FM% calculated using DXA as compared to BMI. The prediction of FM% based on height/waist using linear regression was better when both male and female subjects were considered than when separated by sex. This coincides with the findings of Woolcott & Bergman and could be simpler in its application in the field or in clinical settings.

Compared to BMI, RFM explained 84% of the variability and the RMSE decreased by 50%. We performed the same analysis with our data set, applying the RFM equation and regressed it on DXA. The results were almost identical. This procedure was repeated, changing DXA for ADP, BIA and the 4C model for measuring FM%. Even though ADP, BIA and the 4C model were very similar to DXA in terms of the improvement of precision (Pearson’s r, R^2^ and RMSE), accuracy decreased significantly (intercept different from zero).

The use of WC has been suggested as a measure to evaluate adiposity-related to morbidity and mortality [40–43]. In our study, WC was measured only at the level of the umbilical mark in contrast to the NHANES population, where WC was measured at the level of the uppermost lateral border of the right ilium. In our study, we cannot assert that the anatomical location could affect the calculation of RFM. Nonetheless, a study in overweight adults in Brazil found a difference of 3.2 cm in WC in men, measured at the umbilical level and immediately above the iliac crest, while in women the difference was only 0.1 cm [44]. Also, a study in older adults measured WC at ten different sites in relation to abdominal fat by DXA and found that the association was practically identical [45]. Another study by a panel of experts conducted a systematic review of 120 studies (236 samples) to determine whether measurement protocol influenced the relationship of WC with morbidity of cardiovascular disease (CVD) and diabetes with mortality from all causes [46]. Most of the protocols they reviewed, measured WC at the midpoint, umbilicus or minimal waist. Their findings suggested that WC measurement protocols had no substantial influence on the association between WC, all-case and CVD mortality, CVD and diabetes.

Furthermore, the relationship of WC with other anthropometric measurements like height (which is included as part of the RFM equation), has received a lot of interest due to its association with visceral fat and cardiometabolic risk factors in different populations worldwide, ethnicities and age groups [47–55].

The performance of RFM in estimating FM% by DXA in our subjects was more consistent overall than BMI, and almost identical to that reported by Woolcott and Bergman [11]. The better performance of RFM could be due to the association of WC to abdominal and visceral fat, as reported in many studies [56–59]. In our case we found that WC had a higher correlation with abdominal fat mass from DXA compared to regional fat mass from arms, legs and total fat mass.

A positive aspect of our study was the comparison of RFM and BMI against DXA in a group of adults from north-west Mexico as well as to evaluate the prediction of FM% by RFM against ADP, BIA, and the 4C model since some studies have found that DXA can either underestimate or overestimate FM% [60–65]. In our study of 61 male and female adults, FM% measured by each of ADP, BIA and the 4C model were not significantly different. However, DXA overestimated FM% in comparison to the other three methods by an average of 5.04%.

## Conclusion

This external validation proved that the performance of the RFM equation used in this study to estimate whole body fat percentage was better and more consistent overall than BMI in this Mexican population. Further, RFM showed a stronger correlation with DXA than with the other body composition methods (ADP, BIA and the 4C model). The better performance of RFM could be due to the association of WC to abdominal and visceral fat, as reported in many studies and in our findings. Nevertheless, important limitations of our study were, not having a larger number of subjects, a more representative sample for all the Mexican population, a higher proportion of individuals with overweight and obesity as well as a better description of physical activity. In spite of this, it seems to work adequately for younger and thinner Mexican Mestizos from north-west Mexico.

Due to its simplicity in terms of the anthropometric measurements required (height and WC) the RFM index could be used in daily clinical practice as a tool for the evaluation and monitoring of body composition in Mexican adults.

## Supporting information

S1 File RFM dataset. (XLSX)

S2 File Demographic information dataset. (XLSX)
